# Primary and acquired resistance to immunotherapy in NSCLC

**DOI:** 10.3389/fimmu.2026.1864819

**Published:** 2026-07-09

**Authors:** Bo Yuan, Wenzhi Deng, Juan Luo, Yu Xin, Xiulin Jiang, Xinrong Liu, Quan Cai

**Affiliations:** 1 Department of Spine Surgery, Hunan Aerospace Hospital, The Affiliated Aerospace Hospital of Hunan Normal University, Changsha, Hunan, China; 2 Department of Pathology, The Third Xiangya Hospital of Central South University, Changsha, China; 3 Department of Oncology, Qujing Hospital of Traditional Chinese Medicine, Qujing, China; 4 College of Life Science, University of Chinese Academy of Sciences, Beijing, China; 5 Department of General Surgery, Hunan Aerospace Hospital, The Affiliated Aerospace Hospital of Hunan Normal University, Changsha, Hunan, China

**Keywords:** antigen presentation, biomarkers, combination immunotherapy, epigenetic regulation, immune checkpoint inhibitors, immune resistance, multi-omics analysis immunotherapy resistance, non-small cell lung cancer

## Abstract

Non-small cell lung cancer (NSCLC) is one of the leading causes of cancer incidence and mortality worldwide. In recent years, immune checkpoint inhibitors (ICIs), particularly those targeting the programmed cell death protein 1/programmed death-ligand 1 (PD-1/PD-L1) axis, have significantly improved survival outcomes in a subset of patients. However, the magnitude and durability of clinical benefit vary considerably according to PD-L1 expression, treatment setting, histological subtype, oncogenic driver status, and whether ICIs are administered as monotherapy or in combination regimens. A substantial proportion of patients therefore exhibit either primary resistance or acquired resistance after an initial response. This review systematically summarizes the key mechanisms underlying immune resistance in lung cancer. These include defects in antigen presentation, such as abnormalities in major histocompatibility complex class I (MHC-I), transporter associated with antigen processing 2 (TAP2), and β2-microglobulin (B2M), as well as dysregulation of the interferon-γ/Janus kinase-signal transducer and activator of transcription (IFN-γ/JAK-STAT) signaling pathway. Tumors frequently exhibit an immune-excluded or ‘cold’ phenotype, which further limits immune recognition and reduces responsiveness to immunotherapy. This review summarizes immune resistance in NSCLC through a framework that distinguishes primary resistance from acquired resistance. Primary resistance reflects failure of immune activation at treatment initiation, usually due to pre-existing tumor-intrinsic or microenvironmental barriers, including impaired antigen presentation, defective IFN-γ/JAK-STAT signaling, low tumor immunogenicity, immune-cold or immune-excluded phenotypes, and suppressive TME states. In contrast, acquired resistance reflects adaptive tumor and immune ecosystem evolution under therapeutic pressure, leading to neoantigen loss, HLA or B2M alterations, compensatory checkpoint activation, progressive T cell exhaustion, TME remodeling, and epigenetic stabilization of immune escape. We further discuss mechanism-based biomarkers, translational correlates, and rational therapeutic strategies for overcoming resistance.

## 1 Introduction

Lung cancer remains one of the most common malignancies worldwide and is a leading cause of cancer-related morbidity and mortality, posing a continuous threat to global public health (1). Among its subtypes, NSCLC accounts for approximately 80%–85% of all lung cancer cases, including major histological types such as adenocarcinoma, squamous cell carcinoma, and large cell carcinoma (2). Although early-stage patients may achieve a potential cure through surgery combined with adjuvant therapy, most patients are diagnosed at an advanced stage (3). In these cases, conventional chemotherapy and radiotherapy provide only limited survival benefit.

In recent years, cancer immunotherapy, particularly ICIs, has markedly changed the therapeutic landscape of NSCLC (4). Monoclonal antibodies targeting PD-1, such as nivolumab and pembrolizumab, can block the PD-1/PD-L1 signaling axis and restore the effector function of tumor-specific T cells (5). Durable antitumor immune responses and long-term survival benefits have been observed in a subset of patients (6). This breakthrough has established immunotherapy as a cornerstone treatment for advanced NSCLC and has driven the development of combination strategies and personalized treatment approaches.

However, despite these clinical advances, therapeutic responses to ICIs vary substantially across patient subgroups and treatment contexts. In unselected NSCLC populations, only a minority of patients achieve durable benefit from ICI monotherapy, whereas response rates are generally higher in patients with high PD-L1 expression, in selected first-line settings, and with chemo-immunotherapy or dual-checkpoint combinations. Conversely, responses are often lower in tumors with low or absent PD-L1 expression, oncogenic driver alterations such as EGFR mutations, poor T cell infiltration, or other immune-excluded features (7). Thus, primary resistance reflects failure of immune activation at treatment initiation, typically due to pre-existing tumor-intrinsic or microenvironmental barriers (8, 9). In contrast, acquired resistance reflects adaptive tumor evolution under sustained immune pressure, leading to progressive immune escape after an initial period of clinical benefit (10). These two resistance patterns arise from partially overlapping but biologically distinct mechanisms, including impaired antigen presentation, defective interferon signaling, immune-excluded tumor phenotypes, suppressive TME remodeling, compensatory checkpoint activation, and tumor evolutionary adaptation (11). Understanding these mechanisms is essential for improving patient stratification, optimizing rational combination therapies, and identifying new therapeutic targets (12).

## 2 Overview of the cancer immunity cycle

The antitumor immune response can be described by the Cancer Immunity Cycle, which represents a series of continuous and interdependent steps from tumor recognition to elimination (13). Briefly, the cycle includes: release of tumor antigens after cancer cell death, uptake and processing of antigens by antigen-presenting cells (mainly dendritic cells), presentation of antigens via major histocompatibility complex (MHC) molecules, activation of naïve T cells in lymphoid organs, trafficking and infiltration of effector T cells into tumor sites, and finally recognition and killing of tumor cells (13). This process establishes a positive feedback loop in which tumor cell destruction releases additional antigens, thereby amplifying subsequent immune responses (14). Under ideal conditions, this cycle sustains a strong and continuous antitumor immunity. Nevertheless, in solid tumors such as lung cancer, multiple layers of interference can disrupt this cycle at distinct stages. Low antigenicity, impaired antigen presentation, defective dendritic cell priming, insufficient T cell trafficking, stromal exclusion, and immunosuppressive cytokine or metabolic networks may each interrupt antitumor immunity (15). In contrast, acquired resistance often develops after partial immune activation, when tumor cells and the surrounding immune microenvironment adapt under therapeutic pressure to restore immune escape (16). Therefore, the Cancer Immunity Cycle provides a useful conceptual structure for distinguishing early immune-activation failure from later immune-evolutionary escape in NSCLC (14).

## 3 Primary resistance mechanisms

**Figure 1:**
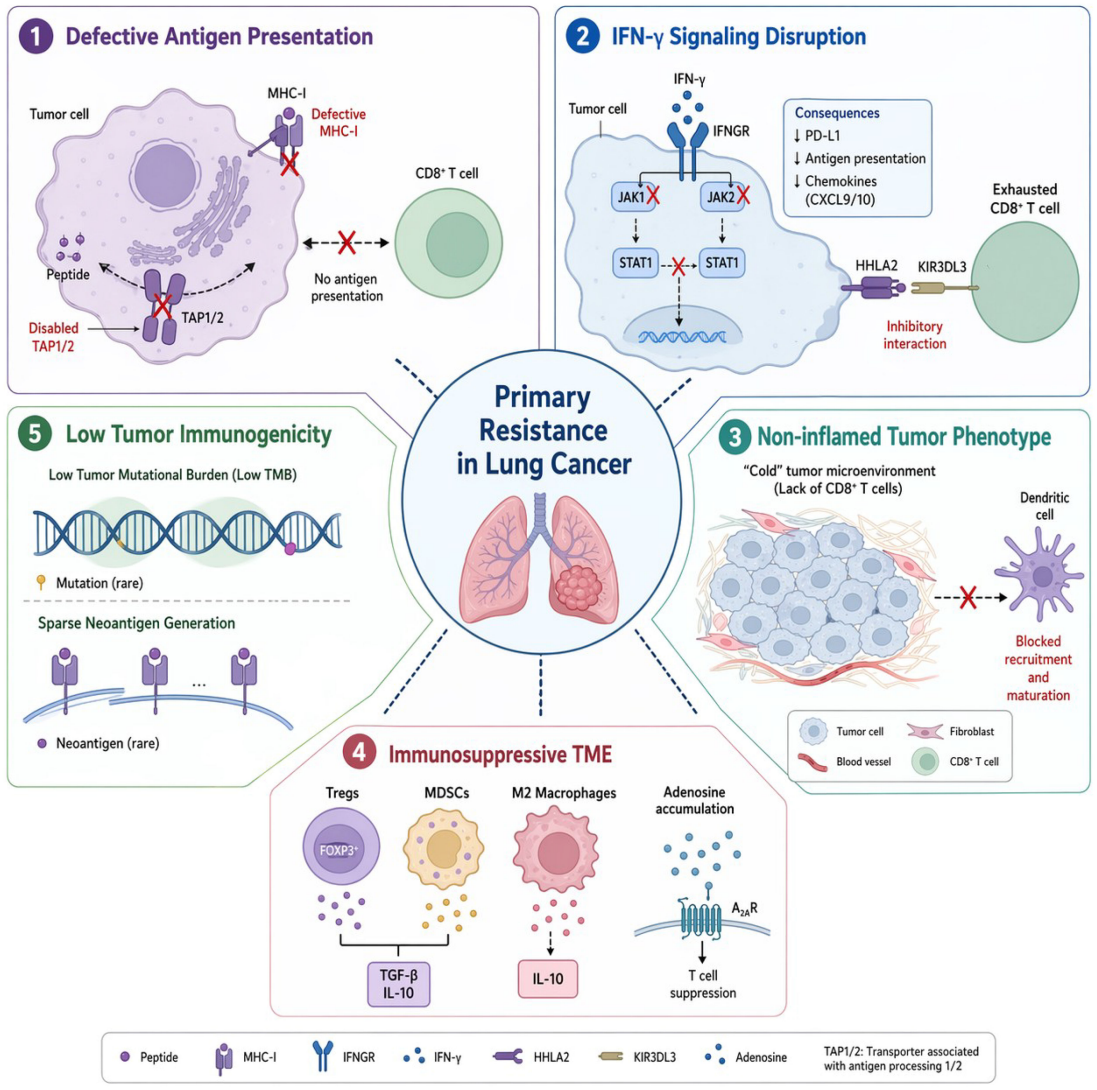
Mechanisms of primary resistance to immune checkpoint blockade in lung cancer. Schematic overview of major mechanisms underlying primary resistance, including defective antigen presentation (e.g., MHC-I/TAP1/2 alterations), impaired IFN-γ signaling (JAK/STAT pathway disruption), non-inflamed (“cold”) tumor microenvironment with poor T cell infiltration, immunosuppressive tumor microenvironment (Tregs, MDSCs, M2 macrophages, adenosine), and intrinsically low tumor immunogenicity characterized by low tumor mutational burden and neoantigen scarcity.

In NSCLC, these defects include impaired antigen presentation, disrupted IFN-γ signaling, immune-excluded tumor architecture, suppressive TME components, and low intrinsic tumor immunogenicity. **Figure 1** summarizes these tumor-intrinsic and tumor-extrinsic mechanisms that limit the initial efficacy of immune checkpoint blockade.

### 3.1 Defective antigen presentation

Defective antigen presentation can contribute to both primary and acquired resistance, but the timing and biological implications differ. These pre-existing defects may include low baseline MHC-I expression, impaired antigen-processing machinery, TAP1/2 downregulation, B2M abnormalities, or inflammatory and epigenetic programs that suppress antigen display. As a result, tumor cells remain poorly visible to CD8+ T cells from the beginning of treatment, thereby preventing the initiation of effective tumor-reactive immunity (17). Recent studies have shown that selective TAP2 downregulation occurs in approximately 42.4% of untreated NSCLC cases and is strongly associated with reduced response to immune checkpoint inhibitors (18). TAP2 loss not only impairs peptide transport and MHC-I antigen presentation but also upregulates SOCS1, thereby inhibiting interferon-related signaling and weakening tumor responsiveness to inflammatory cytokines (18). This ultimately enhances resistance to antigen-specific T cell killing. Beyond this, TAP2 downregulation is associated with reduced chromatin accessibility at its promoter and increased IL-4 signaling, which is mainly derived from tumor-infiltrating myeloid cells. The IL-4/IL-4Rα axis further suppresses TAP2 expression and reinforces an immunosuppressive state (18). Conversely, blockade of this pathway can enhance T cell-mediated tumor killing. Together, TAP2-associated antigen presentation defects and IL-4-driven microenvironmental regulation represent key drivers of primary immune resistance in NSCLC.

### 3.2 IFN-γ signaling disruption

Interferon-γ (IFN-γ) signaling plays a central role in antitumor immunity by regulating antigen presentation, immune cell recruitment, and PD-L1 expression. This pathway mainly depends on the JAK/STAT axis (19). Functional mutations in JAK1 or JAK2 can disrupt IFN-γ signaling and reduce tumor responsiveness to immune stimulation (19). Abnormalities in downstream transcription factors such as STAT1 further impair the expression of immune-related genes. These alterations weaken antigen presentation and reduce immune cell infiltration, thereby promoting immune escape (20). Tumor cells with high expression of HHLA2 can bind to the inhibitory receptor KIR3DL3 on T cells, inducing CD8^+^ T cell exhaustion (21). Mechanistically, HHLA2-KIR3DL3 signaling suppresses glutamine transport and metabolism through the ERK/MAPK pathway, leading to metabolic insufficiency and functional exhaustion of T cells. This results in decreased effector cytokines such as IFN-γ and TNF-α, along with increased IL-10 production, forming a metabolically driven exhausted T cell state. Some NSCLC and small cell lung cancers also exhibit intrinsic resistance to IFN-γ signaling, which reduces responsiveness to PD-1/PD-L1 blockade. Certain tumors lose IFN-γ responsiveness due to inactivation of JAK2 or IFNGR1, while others show a low-responder phenotype characterized by persistently low expression of interferon-stimulated genes. This is often accompanied by reduced H3K27 acetylation, promoter hypermethylation, and impaired IRF1 recruitment. In neuroendocrine lung cancer, MYC amplification further suppresses JAK2 expression, weakening IFN-γ pathway activation and reducing immunotherapy efficacy.

### 3.3 Non-inflamed tumor phenotype

The non-inflamed or “immune-cold” tumor phenotype is a hallmark of primary resistance and is characterized by minimal or absent CD8+ T cell infiltration within both the tumor parenchyma and surrounding stroma (22). This phenotype differs from the “immune-excluded” phenotype, in which immune cells are present at the invasive margin or stromal compartment but fail to penetrate the tumor nests. Thus, immune-cold tumors primarily reflect insufficient immune priming, poor antigenicity, or defective T cell recruitment, whereas immune-excluded tumors often reflect physical, stromal, vascular, or cytokine-mediated barriers that prevent effector T cells from entering tumor cell regions (23). At the molecular level, aberrant activation of the WNT/β-catenin pathway is more closely associated with immune-cold tumors by suppressing dendritic cell recruitment and T cell priming (23). By contrast, TGF-β signaling, CAF activation, abnormal vasculature, and extracellular matrix remodeling are frequently linked to immune exclusion by limiting T cell penetration into tumor nests (24). Loss of the tumor suppressor PTEN activates the PI3K-AKT pathway, promoting immunosuppressive factor expression and reducing T cell infiltration (25). Because these tumors lack pre-existing immune activity, they are generally less responsive to immune checkpoint inhibitors.

### 3.4 Immunosuppressive tumor microenvironment

Within the tumor microenvironment (TME), Tregs, MDSCs, and M2-like TAMs cooperate with suppressive cytokines, adenosine signaling, and metabolic constraints to inhibit effector T cell activity. TGF-β and IL-10 directly reduce CD8+ T cell proliferation and cytotoxicity, whereas the CD39/CD73–adenosine axis suppresses T cell receptor signaling. At the metabolic level, glucose competition, lactate accumulation, and extracellular acidification further impair T cell function and promote exhaustion (26). TGF-β signaling not only suppresses T cell infiltration but also contributes to extracellular matrix remodeling and the formation of immune-excluded tumor structures (27). Together, these mechanisms—cellular suppression, cytokine networks, and metabolic constraints—create a highly inhibitory immune environment and represent a major barrier to effective immunotherapy.

### 3.5 Low tumor immunogenicity

Low tumor immunogenicity is another important cause of primary resistance. A low tumor mutational burden (TMB) leads to fewer neoantigens and reduces the likelihood of immune recognition (28). In NSCLC, certain driver mutations such as EGFR are typically associated with low TMB and a “cold tumor” phenotype, resulting in significantly lower response rates to immune checkpoint inhibitors (29). For example, the transcription factor TCF1 is essential for the initial activation and expansion of tumor antigen-specific CD8^+^ T cells in low-immunogenic tumors (30). Nevertheless, in highly immunogenic tumors, effector T cell expansion can compensate for reduced dependence on TCF1 (31). Importantly, enhancing antigen presentation or using vaccination strategies can restore T cell priming capacity even in TCF1-deficient contexts, thereby partially rescuing the efficacy of immune checkpoint blockade.

## 4 Acquired resistance mechanisms

**Figure 2:**
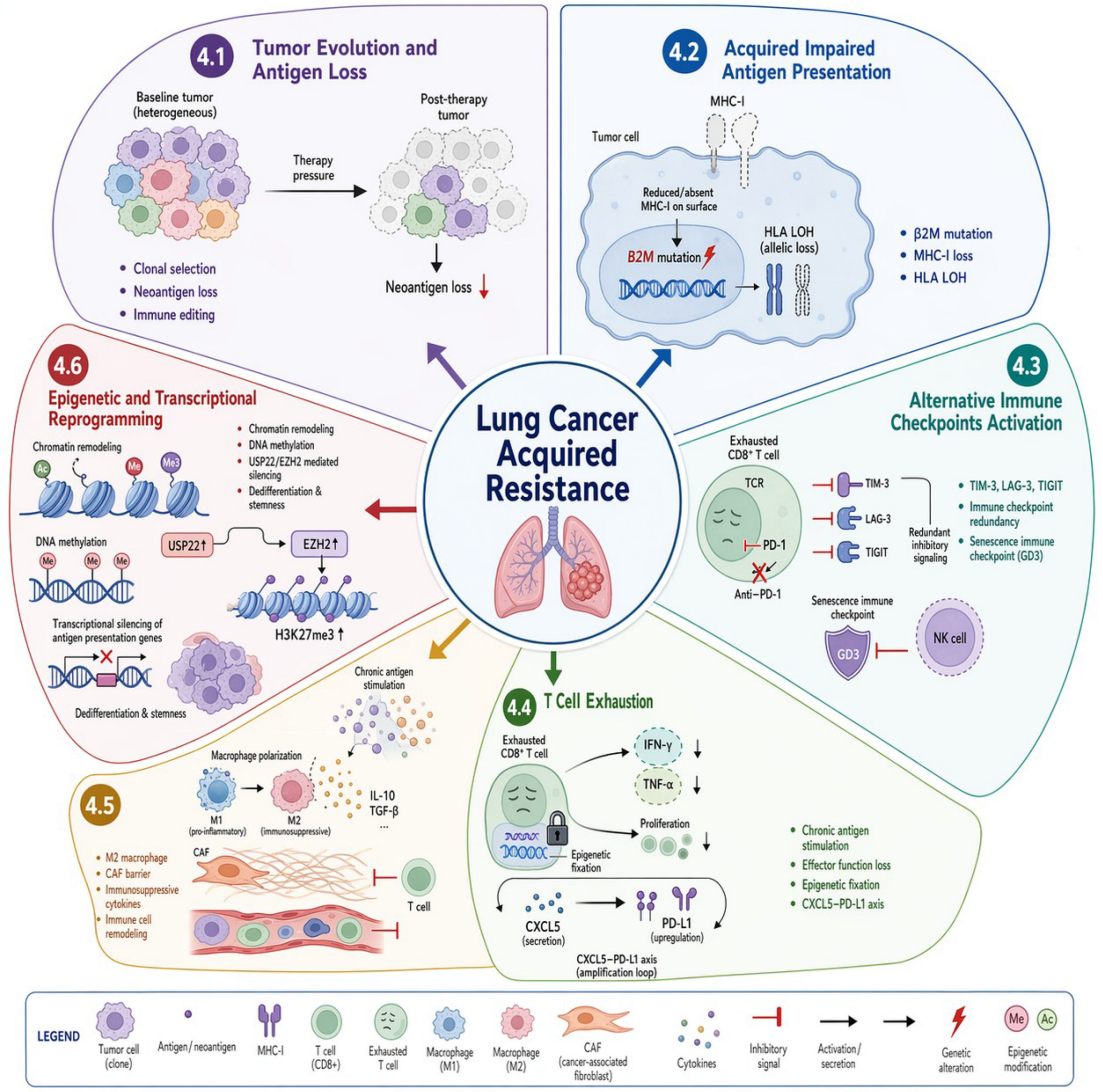
Mechanisms of acquired resistance to immune checkpoint blockade in lung cancer. Overview of key pathways driving acquired resistance following therapy, including tumor evolution and immune editing with neoantigen loss, acquired defects in antigen presentation (e.g., B2M mutation, HLA loss), upregulation of alternative immune checkpoints (TIM-3, LAG-3, TIGIT), T cell exhaustion, and epigenetic/transcriptional reprogramming. Additional contributions include immunosuppressive stromal remodeling and myeloid cell–mediated immune suppression. These mechanisms should be interpreted as interconnected manifestations of immune-driven evolutionary selection rather than independent resistance events.

Acquired resistance is best understood as an immune-driven evolutionary process rather than a collection of isolated molecular events (23). During the initial response to ICIs, immune-sensitive tumor clones are preferentially eliminated, whereas pre-existing or newly emerging subclones with reduced antigenicity, impaired antigen presentation, enhanced immune checkpoint redundancy, or increased stress tolerance gain a selective advantage. At the same time, persistent immune pressure reshapes the surrounding TME by promoting T cell exhaustion, suppressive myeloid remodeling, stromal adaptation, and epigenetic plasticity (32). Therefore, mechanisms such as neoantigen loss, HLA or B2M alterations, alternative checkpoint activation, T cell exhaustion, and epigenetic reprogramming should be viewed as interconnected manifestations of therapy-imposed evolutionary selection. **Figure 2** illustrates how these tumor-intrinsic and tumor-extrinsic adaptations cooperate to erode durable immunotherapy efficacy.

### 4.1 Tumor evolution and antigen loss

Under immune selection pressure, tumor cells undergo clonal selection and evolutionary remodeling (33). Highly immunogenic subclones are eliminated, while low-immunogenic variants preferentially survive and expand. This leads to the emergence of immune-escape dominant populations (33). A key event in this process is neoantigen loss, which includes deletion of antigen-encoding mutations or downregulation of their expression. T cell recognition is significantly reduced (33). This process is a classical example of immunoediting, in which tumors transition from an immune elimination phase to an immune escape phase under continuous immune surveillance. Thus, neoantigen loss is not merely a passive reduction in tumor immunogenicity, but a direct consequence of immune editing, in which antigen-positive and highly immunogenic clones are selectively eliminated while antigen-low variants expand under therapeutic pressure (34).

### 4.2 Acquired impairment of antigen presentation

In contrast to primary antigen-presentation defects, acquired antigen-presentation defects emerge or become enriched after an initial response to immunotherapy (35). Under sustained immune pressure, tumor clones that retain antigen presentation are preferentially eliminated, whereas clones with B2M mutations, HLA class I loss, HLA loss of heterozygosity, or impaired antigen-processing machinery gain a selective advantage (35). These alterations allow tumors that were initially recognized by CD8+ T cells to progressively lose immune visibility, leading to disease progression despite prior sensitivity to ICIs. One common mechanism is mutation of β2-microglobulin (B2M), which destabilizes or completely disrupts the MHC-I complex (36). Loss of heterozygosity (LOH) or reduced expression of HLA class I molecules can also occur (36). This study shows that acquired resistance to PD-1/PD-L1 blockade in lung cancer can arise from disruption of HLA class I antigen processing, particularly through loss of B2M, leading to impaired antigen presentation and immune escape (36). Functional validation in mouse models confirmed that B2m loss confers resistance to immune checkpoint inhibitors *in vivo* (36). These alterations allow tumor cells that were initially detectable by the immune system to gradually lose CD8^+^ T cell recognition. Consequently, tumor cells regain an immune-evading state even after an initial response to immunotherapy. From an evolutionary perspective, HLA loss, B2M mutation, and impaired antigen-processing machinery provide a strong selective advantage because they allow tumor cells to retain malignant fitness while escaping CD8+ T cell recognition. These alterations therefore represent a convergent route of immune evasion selected during ongoing checkpoint blockade. Therefore, primary antigen-presentation defects mainly explain why tumors fail to respond at treatment initiation, whereas acquired defects explain how initially responsive tumors escape renewed immune attack during therapy. Distinguishing these two scenarios is important because they may require different therapeutic strategies and different biomarker approaches.

### 4.3 Alternative immune checkpoint activation

Under selective pressure from checkpoint blockade, tumors may maintain immune suppression by activating alternative inhibitory pathways, including TIM-3, LAG-3, and TIGIT (37). These receptors can function independently or cooperatively to suppress T cell activity, creating pathway redundancy that sustains immune escape despite checkpoint inhibition and promotes progressive T cell exhaustion (37). In parallel, senescent cells can upregulate the surface molecule disialylated ganglioside GD3, which helps them evade immune clearance (38). GD3 is elevated in multiple aging-related tissues, including lung, liver, kidney, and bone, and creates an “immune-privileged” state by suppressing natural killer (NK) cell function. Importantly, targeting GD3 can eliminate GD3^+^ senescent cells and reduce fibrosis and tissue remodeling in experimental models (38). Therefore, GD3 has been proposed as a senescence-associated immune checkpoint (senescence immune checkpoint, SIC), representing a novel mechanism of immune evasion. The emergence of alternative inhibitory receptors can therefore be interpreted as checkpoint diversification under selective pressure. When the PD-1/PD-L1 axis is therapeutically blocked, tumor and immune ecosystems may shift toward other inhibitory pathways, allowing immune suppression to persist through functionally redundant mechanisms.

### 4.4 T cell exhaustion

Chronic antigen exposure is a central driver of T cell exhaustion. During acquired resistance, CD8^+^ T cells are continuously exposed to tumor antigens and inhibitory signals in the tumor microenvironment (39). Over time, they progressively lose effector functions, including reduced production of cytokines such as IFN-γ and TNF-α, decreased proliferation, and weakened cytotoxic activity. Importantly, this exhausted state is stabilized by epigenetic programming and becomes difficult to fully reverse even after antigen removal, which limits long-term therapeutic efficacy (40). In chronic infection models, combined PD-1 blockade and IL-2 therapy can reprogram PD-1^+^TCF1^+^ stem-like CD8^+^ T cells into highly functional effector cells. This effect depends on high-affinity IL-2 receptor signaling (CD25/CD122/CD132), suggesting that stem-like exhausted T cells are not irreversibly fixed and can be reactivated under appropriate stimulation (41). In lung cancer, CXCL5 is highly expressed and is associated with poor prognosis. It activates the Paxillin/AKT signaling pathway to upregulate PD-L1, forming a positive feedback loop that promotes immune suppression (42). CXCL5 recruits neutrophils and further impairs CD8^+^ T cell function, thereby accelerating T cell exhaustion. Combined blockade of CXCL5 and PD-L1 significantly suppresses tumor growth, highlighting CXCL5 as a key link between immunosuppression and immune escape (42). T cell exhaustion during acquired resistance also reflects immune-driven evolution at the cellular ecosystem level. Persistent antigen exposure, chronic inflammatory signaling, metabolic stress, and repeated checkpoint engagement progressively select for dysfunctional T cell states that are less responsive to PD-1 blockade alone (43).

### 4.5 Tumor microenvironment remodeling

Acquired resistance is also accompanied by dynamic remodeling of the tumor microenvironment (TME). This remodeling includes both mechanistic changes within immune and stromal compartments and clinical or translational evidence showing that TME states can be associated with treatment response or resistance.

#### 4.5.1 Mechanistic remodeling of the TME

Acquired resistance is accompanied by coordinated remodeling of immune, stromal, vascular, and metabolic compartments within the TME (44). Tumor-associated macrophages may shift toward an M2-like phenotype and secrete IL-10, TGF-β, and other suppressive mediators that inhibit cytotoxic T cell function (45). MDSCs and Tregs further reinforce immune suppression through arginine depletion, adenosine signaling, cytokine release, and direct inhibition of effector T cells (46). In parallel, CAF expansion and extracellular matrix deposition create physical barriers that restrict T cell penetration into tumor nests, thereby converting an initially inflamed tumor into an immune-excluded state (47). Metabolic constraints, including glucose deprivation, lactate accumulation, hypoxia, and extracellular acidification, further reduce T cell fitness and promote exhaustion (48–50). Therefore, TME remodeling should be viewed not as a single pathway, but as a coordinated ecosystem-level adaptation that protects resistant tumor clones from renewed immune attack.

#### 4.5.2 Clinical and translational correlates of TME remodeling

Clinical and single-cell studies support the relevance of TME remodeling to immunotherapy response in NSCLC. Tumors achieving major pathological response often show enhanced antigen-presentation programs, including activation of MHC class II-related pathways, and enrichment of immune cell populations associated with productive antitumor immunity, such as FCRL4+FCRL5+ memory B cells and CD16+CX3CR1+ monocytes (51). In contrast, non-MPR tumors may display metabolic or hormone-associated programs, including increased estrogen metabolism and elevated serum estradiol, suggesting that non-immune tumor-intrinsic states can shape immune resistance (38). Across treatment contexts, expansion of cytotoxic T cells and CD16+ NK cells, reduction of Tregs, and partial macrophage reprogramming are associated with more favorable immune remodeling (51). These findings indicate that TME features can serve not only as mechanistic drivers of resistance, but also as translational correlates for patient stratification and response monitoring.

#### 4.5.3 Therapeutic implications of TME remodeling

Therapeutic modulation of the TME may provide opportunities to overcome resistance when the dominant barrier is immune exclusion or myeloid/stromal suppression. For example, dasatinib has been reported to increase infiltration of CD4+ and CD8+ T cells, NK cells, M1-like macrophages, and CD11c+ antigen-presenting cells, while reducing Tregs and M2-like macrophages (52). In addition, IL-1β may exert context-dependent effects in NSCLC. Under selected immune conditions, IL-1β can promote CXCL10 secretion by tumor cells, enhance CD8+ T cell recruitment, and restore antitumor immunity through TXNIP-mediated mitochondrial DNA release and AIM2 inflammasome activation (52). These examples suggest that TME-directed strategies should be selected according to the dominant suppressive program rather than applied empirically. In future studies, paired baseline and on-treatment biopsies, spatial immune profiling, and myeloid/stromal biomarkers may help identify patients most likely to benefit from TME-reprogramming combinations.

### 4.6 Epigenetic and transcriptional reprogramming

Epigenetic and transcriptional reprogramming represents a late and stabilizing layer of acquired resistance. Under persistent immune pressure, tumor cells may undergo chromatin remodeling, DNA methylation changes, abnormal histone modifications, and RNA modification-dependent regulation, leading to durable suppression of antigen presentation, innate immune activation, and immune sensitivity (53–56). Unlike transient adaptive signaling, these changes can stabilize resistant phenotypes even after the original immune pressure is reduced, thereby converting reversible immune escape into a more fixed and difficult-to-reverse state. In parallel, tumor cells often acquire dedifferentiation and increased stemness, leading to more plastic and therapy-resistant states that facilitate immune escape and acquired resistance (56). In NSCLC patients who fail immune checkpoint therapy, a key upstream regulator of antigen presentation defects is the deubiquitinase USP22. USP22 stabilizes the histone methyltransferase EZH2, which promotes transcriptional silencing of MHC-I-related genes (57). This suppresses neoantigen presentation and reduces CD8^+^ T cell recognition and killing ability. Genetic or pharmacological inhibition of USP22 can restore MHC-I expression, increase tumor immunogenicity, and reverse resistance to anti-PD-1 therapy (57). These effects are dependent on the EZH2 axis. Clinically, high USP22 expression is associated with low β2M levels, reduced CD8^+^ T cell infiltration, and poor response to ICIs. SMARCA4 deficiency in NSCLC leads to widespread remodeling of SWI/SNF-dependent enhancer landscapes (58). This results in downregulation of innate immune genes such as STING1, IL-1β, and type I interferon signaling pathways, thereby reducing dendritic cell and CD4^+^ T cell infiltration and weakening anti-PD-1 efficacy. Furthermore, the PD-1 encoding gene PDCD1 is regulated by m6A RNA modification. METTL14 mediates PDCD1 mRNA destabilization through YTHDF1/2/3-dependent pathways, thereby reducing PD-1 expression and enhancing CD8^+^ T cell activity (59). Nevertheless, heterozygous loss of METTL14 impairs CD8^+^ T cell activation, increases PD-1 levels, and promotes tumor progression. Clinical data further demonstrate a negative correlation between METTL14 expression and PDCD1 levels across multiple cancers, and its association with resistance to anti-PD-1 immunotherapy. Thus, epigenetic and transcriptional reprogramming should not be viewed merely as one additional resistance mechanism, but as a phenotype-stabilizing process that consolidates multiple immune-evasive traits and may explain why some tumors fail to regain immunotherapy sensitivity even after changes in immune pressure or treatment strategy.

### 4.7 Acquired resistance as immune-driven evolutionary selection

Taken together, the mechanisms described above are highly interconnected and should be interpreted within an immune-driven evolutionary framework. Neoantigen loss reduces tumor visibility; HLA or B2M alterations prevent antigen display; alternative checkpoint pathways restore inhibitory signaling after PD-1/PD-L1 blockade; exhausted T cell states weaken immune pressure; suppressive TME remodeling creates protective niches; and epigenetic reprogramming enables rapid phenotypic adaptation. These events do not necessarily occur in a fixed linear order. Instead, they may arise in parallel or sequentially, depending on pre-existing tumor heterogeneity, treatment intensity, immune infiltration, stromal architecture, and metabolic constraints. This evolutionary view explains why acquired resistance is often heterogeneous across patients and even across lesions within the same patient. It also supports the need for longitudinal monitoring, such as serial biopsies, ctDNA-based clonal tracking, and dynamic immune profiling, to detect emerging escape routes and guide adaptive therapeutic strategies.

## 5 Biomarkers of resistance

Biomarkers of immunotherapy resistance in NSCLC should be interpreted within a clinically relevant framework rather than simply categorized by sample source. From a treatment-decision perspective, three major biomarker groups are particularly important: biomarkers of tumor immunogenicity, biomarkers of immune activation, and dynamic biomarkers that reflect tumor evolution during therapy. This framework better captures how biomarkers are used to estimate baseline immune sensitivity, evaluate the functional immune state of the tumor microenvironment, and monitor emerging resistance over time.

### 5.1 Biomarkers of tumor immunogenicity

Biomarkers of tumor immunogenicity reflect whether tumor cells are likely to generate and present recognizable antigens (60). Tumor mutational burden (TMB) is one of the most widely studied markers in this category because a higher mutation load may increase neoantigen availability and improve the likelihood of immune recognition (61). However, TMB alone is insufficient to predict response, because tumors with high neoantigen burden may still resist immunotherapy if antigen presentation is impaired or if the tumor microenvironment is strongly suppressive (61). Conversely, tumors with low TMB, such as those harboring certain oncogenic driver alterations including EGFR mutations, often display reduced immunogenicity and lower responsiveness to immune checkpoint inhibitors (62). Antigen-presentation capacity is another key component of tumor immunogenicity. Baseline expression of major histocompatibility complex class I (MHC-I), β2-microglobulin (B2M), transporter associated with antigen processing 1/2 (TAP1/2), and HLA status may help identify tumors with intrinsic defects in immune visibility (63). These biomarkers are particularly relevant for distinguishing tumors that lack recognizable antigens from those that possess antigens but fail to present them effectively to CD8+ T cells.

### 5.2 Biomarkers of immune activation

Biomarkers of immune activation indicate whether an antitumor immune response has already been initiated. PD-L1 expression remains a clinically used biomarker for selecting patients for certain immune checkpoint inhibitor regimens, but its predictive value is limited by spatial heterogeneity, temporal variation, assay differences, and dependence on inflammatory signaling (64). Therefore, PD-L1 should be interpreted together with broader immune-context markers rather than as a stand-alone predictor (64). The density, localization, and functional state of tumor-infiltrating lymphocytes are also important. High CD8+ T cell infiltration and interferon-γ (IFN-γ)-related gene signatures generally reflect a pre-existing inflamed tumor microenvironment and may be associated with better response to ICIs. However, these markers must be interpreted carefully. In immune-cold tumors, T cell infiltration is minimal or absent, whereas in immune-excluded tumors, T cells are present at the tumor margin or stromal compartment but fail to enter tumor nests. These two phenotypes may both show resistance to ICIs but require different therapeutic strategies. Additional immune-activation markers, including chemokine signatures, dendritic cell activity, T cell receptor clonality, and exhaustion-associated markers, may further improve response prediction when integrated with PD-L1 and TMB.

### 5.3 Dynamic biomarkers

Dynamic biomarkers are essential for monitoring acquired resistance because immune resistance evolves during treatment. Circulating tumor DNA (ctDNA) provides a non-invasive readout of tumor burden, clonal evolution, and emerging resistance alterations (65). Clearance of ctDNA during therapy may correlate with treatment response, whereas rising ctDNA or newly detected resistant clones may indicate early disease progression before radiographic changes become apparent (66). Longitudinal tissue or blood-based monitoring can also identify therapy-emergent antigen-presentation defects, such as B2M mutation, HLA loss, or reduced MHC-I expression at progression. Circulating cytokines, including interleukin-6 (IL-6), IFN-γ, and transforming growth factor-β (TGF-β), may reflect systemic inflammatory or immunosuppressive states, although their standalone predictive value remains limited because of temporal fluctuation and multiple cellular origins (67). Therefore, dynamic biomarkers are most useful when interpreted together with baseline tumor immunogenicity and immune activation markers. An integrated biomarker strategy combining TMB, PD-L1, antigen-presentation status, immune infiltration, IFN-γ signatures, spatial immune architecture, and ctDNA kinetics may better guide patient stratification, early resistance detection, and adaptive therapeutic adjustment. Because resistance to ICIs in NSCLC involves multiple tumor-intrinsic and tumor-extrinsic mechanisms, individual biomarkers are unlikely to fully predict therapeutic response. A mechanism-based framework linking resistance pathways, candidate biomarkers, and potential therapeutic interventions may therefore help guide rational patient stratification and combination treatment design. **Table 1** summarizes major resistance mechanisms, associated biomarkers, and potential therapeutic strategies in NSCLC immunotherapy.

**Table 1:** Resistance mechanisms, associated biomarkers, and potential therapeutic interventions in NSCLC immunotherapy.

Resistance mechanism	Associated biomarkers	Potential therapeutic interventions	Ref
Defective antigen presentation	Low or lost MHC-I expression; B2M mutation/loss; TAP1/2 downregulation; HLA loss of heterozygosity	Epigenetic therapy to restore antigen presentation; IFN pathway activation; cancer vaccines; adoptive T cell therapy; combination with ICIs	(17, 77)
IFN-γ/JAK-STAT signaling disruption	JAK1/2 mutation; IFNGR1 alteration; low STAT1/IRF1 activity; reduced interferon-stimulated gene signatures	Alternative immune activation strategies; STING agonists; cytokine-based therapy; combination immunotherapy independent of IFN-γ signaling	(78, 79)
Immune-cold phenotype	Low CD8+ T cell infiltration; low chemokine expression; WNT/β-catenin activation; EGFR mutation; low inflammatory gene signatures	DC activation; cancer vaccines; radiotherapy; chemo-immunotherapy; oncolytic viruses; strategies enhancing T cell priming and recruitment	(80)
Immune-excluded phenotype	T cells restricted to tumor margin or stroma; high TGF-β signaling; CAF activation; dense extracellular matrix; abnormal vasculature	TGF-β blockade; CAF/stromal targeting; anti-angiogenic therapy; ECM remodeling; combination with ICIs	(81, 82)
Immunosuppressive TME	Increased Tregs, MDSCs, and M2-like TAMs; high IL-10, TGF-β, CD39/CD73, adenosine; lactate accumulation	Treg/MDSC/TAM-targeting strategies; CSF1R, CCR2/CXCR2, CD73/A2A receptor blockade; metabolic intervention; combination immunotherapy	(83)
Low tumor immunogenicity	Low TMB; low neoantigen burden; EGFR or ALK alterations; weak antigen-specific T cell responses	Chemo-immunotherapy; radiotherapy; cancer vaccines; neoantigen-based therapy; strategies increasing antigen release and presentation	(28)
Tumor evolution and neoantigen loss	Loss of clonal neoantigens; reduced TCR clonality; ctDNA-based clonal evolution; immune editing signatures	Dynamic ctDNA monitoring; adaptive treatment adjustment; multi-antigen vaccines; adoptive cellular therapy; combination regimens targeting multiple immune escape routes	(33)
Alternative checkpoint activation	Increased TIM-3, LAG-3, TIGIT, CTLA-4, HHLA2/KIR3DL3, or other inhibitory receptors	Dual or multi-checkpoint blockade; PD-1/PD-L1 plus CTLA-4, LAG-3, TIGIT, or novel checkpoint inhibitors	(84)
T cell exhaustion	High PD-1, TIM-3, LAG-3, TOX; loss of CD28; reduced IFN-γ/TNF-α; exhausted T cell transcriptional signatures	PD-1-based combinations; IL-2 or IL-15-based strategies; metabolic reprogramming; adoptive T cell therapy; reversal of exhaustion-associated epigenetic states	(85)
Epigenetic and transcriptional reprogramming	High USP22/EZH2; SMARCA4 loss; altered DNA methylation or histone modification; abnormal m6A regulators such as METTL14	EZH2 or USP22 targeting; epigenetic therapy; STING pathway activation; RNA modification-targeted therapy; combination with ICIs	(57)
Metabolic immune suppression	High lactate; glucose competition; hypoxia signatures; adenosine pathway activation; altered mitochondrial fitness	LDHA/MCT targeting; adenosine-axis blockade; hypoxia modulation; mitochondrial/metabolic support for T cells; combination with ICIs	(86)
Acquired TME remodeling	Increased CAFs, M2-like TAMs, Tregs, MDSCs; reduced effector T cell infiltration; stromal exclusion signatures	Myeloid reprogramming; stromal targeting; anti-angiogenic therapy; TGF-β blockade; adaptive combination therapy	(87)

## 6 Therapeutic strategies to overcome immune resistance

Mechanistic insights into resistance have supported the development of combination strategies designed to restore antigen visibility, reinvigorate T cell function, and remodel suppressive immune niches. When interpreting clinical trials of combination immunotherapy, response rates should not be viewed as isolated numerical outcomes. They must be interpreted according to treatment line, prior exposure to ICIs, PD-L1 status, histology, tumor immune phenotype, patient selection, and whether the regimen is used to overcome primary resistance or acquired resistance. A low ORR in a post-PD-(L)1 failure setting may indicate that simply adding another checkpoint inhibitor is insufficient once tumors have developed antigen-presentation defects, terminal T cell exhaustion, alternative suppressive pathways, or stable epigenetic resistance programs.

### 6.1 Combination immunotherapy

Dual-checkpoint blockade is designed to enhance antitumor immunity at complementary stages of the immune response. CTLA-4 blockade primarily promotes early T cell priming and clonal expansion, whereas PD-1 blockade mainly restores effector function within peripheral tissues and the TME (68). In selected first-line NSCLC settings, nivolumab plus ipilimumab has shown higher and more durable response rates than PD-1 blockade alone, supporting the value of dual-checkpoint inhibition when endogenous antitumor immunity remains recoverable (69). However, this benefit must be balanced against increased immune-related toxicity. In contrast, the activity of durvalumab plus tremelimumab after prior PD-(L)1 failure appears limited, with low objective response rates in refractory or relapsed patients (70). This difference highlights an important biological distinction: dual-checkpoint blockade may deepen an existing immune response in treatment-naïve or selected patients, but empiric checkpoint escalation may be insufficient once tumors have developed antigen-presentation defects, terminal T cell exhaustion, suppressive myeloid remodeling, or epigenetically stabilized immune escape. Therefore, combination immunotherapy should be guided by resistance mechanisms and treatment context rather than by checkpoint addition alone.

### 6.2 Chemo-immunotherapy

Chemotherapy can enhance immunotherapy efficacy by inducing immunogenic cell death, increasing antigen release, promoting dendritic cell-mediated antigen presentation, and reducing suppressive populations such as Tregs and MDSCs (65). Through these mechanisms, chemotherapy may convert poorly inflamed tumors into a more immune-responsive state and has therefore become a major first-line partner for ICIs in NSCLC (71). Importantly, the benefit of chemo-immunotherapy is most likely when chemotherapy restores antigen availability and inflammatory priming before irreversible immune escape has developed (72). Emerging strategies in other thoracic malignancies, such as bispecific T cell engagers combined with PD-L1 blockade in extensive-stage SCLC, further suggest that adding a new immune effector mechanism may be more effective than simply intensifying PD-1/PD-L1 blockade (73). However, such approaches require histology-specific validation before extrapolation to NSCLC.

### 6.3 Radio-immunotherapy

Radiotherapy exerts local cytotoxic effects while also enhancing systemic antitumor immunity by inducing DNA damage, promoting antigen release, activating type I interferon signaling, and upregulating MHC-I expression (74). These immune-stimulatory effects may support the abscopal response, in which non-irradiated lesions also regress, and provide a biological rationale for combining radiotherapy with immunotherapy. For example, YTHDF2 has been identified as a key regulator of myeloid-derived suppressor cells (MDSCs) in the radiation-induced tumor microenvironment. Ionizing radiation induces MDSC expansion in both mouse and human tumors and increases YTHDF2 expression. In myeloid cells, YTHDF2 enhances MDSC differentiation, infiltration, and suppressive function (75). Conditional deletion of Ythdf2 in myeloid cells significantly weakens MDSC-mediated immunosuppression, enhances antitumor immunity, and reverses radiotherapy resistance. Mechanistically, radiation-induced YTHDF2 expression depends on NF-κB signaling. In turn, YTHDF2 further enhances NF-κB activity by degrading mRNAs of its negative regulators, forming a positive feedback loop (“IR–YTHDF2–NF-κB”) that sustains immunosuppression (75). Inhibition of YTHDF2 reduces MDSC activity and enhances the synergistic effect of radiotherapy combined with anti-PD-L1 therapy. Limitations of radio-immunotherapy include tumor hypoxia, which reduces DNA damage efficacy, and radiation-induced PD-L1 upregulation, which promotes immune resistance. To address these challenges, a mitochondria-targeted nanoplatform (TPP-LND@Lip) has been developed. This system combines triphenylphosphonium (TPP^+^) and lonidamine (LND) in a liposomal formulation. TPP-LND@Lip activates AMPK signaling, downregulates PD-L1 expression, and reverses tumor hypoxia (76). These findings suggest that radio-immunotherapy may be most effective when radiotherapy increases antigen release and interferon signaling without simultaneously amplifying suppressive myeloid feedback. Therefore, radiation dose, fractionation, timing relative to ICIs, tumor hypoxia, and baseline myeloid composition are likely to determine whether radiotherapy functions as an immune sensitizer or instead promotes adaptive immune resistance. Strategies that reduce hypoxia, limit radiation-induced myeloid suppression, or prevent adaptive PD-L1 upregulation may improve the therapeutic window of radio-immunotherapy (76).

### 6.4 Critical interpretation of clinical evidence

Clinical evidence should be interpreted according to treatment setting, prior ICI exposure, PD-L1 status, histology, and the dominant resistance mechanism. Regimens that improve outcomes in treatment-naïve or biomarker-selected patients may show limited activity after established ICI resistance, because the biology has shifted from immune activation failure to an evolved resistant ecosystem. Therefore, future trials should move beyond empirical combination therapy and incorporate mechanism-based patient selection, paired baseline and progression biopsies, ctDNA-based clonal tracking, and immune-monitoring endpoints. Such designs may better identify whether a given strategy restores antigen presentation, reverses immune exclusion, targets suppressive myeloid or stromal programs, or overcomes epigenetically stabilized immune escape.

## 7 Future perspectives

### 7.1 Emerging technologies for resistance profiling

The future of NSCLC immunotherapy will likely shift from static biomarker-based stratification toward integrated and dynamic predictive systems. Multi-omics integration, including genomic, transcriptomic, epigenomic, proteomic, and metabolomic profiling, may provide a more comprehensive view of tumor antigenicity, antigen presentation capacity, immune cell states, and microenvironmental constraints. Single-cell sequencing can further resolve intratumoral heterogeneity by identifying rare resistant clones, exhausted T cell subsets, suppressive myeloid populations, and therapy-induced immune remodeling that may be masked in bulk analyses.

### 7.2 Barriers to clinical implementation

However, several limitations currently restrict the clinical translation of these technologies. Single-cell sequencing is often limited by tissue availability, sampling bias, dissociation-induced artifacts, loss of spatial information, high cost, and the need for specialized computational expertise. Spatial transcriptomics helps preserve tissue architecture and can distinguish immune-inflamed, immune-cold, and immune-excluded phenotypes by mapping immune cells across tumor nests, stromal regions, and invasive margins. Nevertheless, current spatial platforms differ in resolution, sensitivity, throughput, and compatibility with formalin-fixed clinical specimens. Many datasets also remain cross-sectional rather than longitudinal, making it difficult to determine whether observed spatial patterns are causes or consequences of treatment resistance.

Artificial intelligence-based models may integrate imaging, pathology, multi-omics, spatial data, and longitudinal clinical variables to predict response and detect early resistance. Yet clinical implementation remains challenging. Major barriers include small and heterogeneous training cohorts, lack of external validation, batch effects across platforms and institutions, limited interpretability, potential algorithmic bias, regulatory requirements, and difficulties in integrating complex outputs into routine clinical decision-making. Therefore, although single-cell sequencing, spatial transcriptomics, and AI provide powerful tools for understanding NSCLC immunotherapy resistance, their clinical value will depend on standardized workflows, prospective validation, cost-effective assays, reproducible biomarkers, and clinically interpretable decision frameworks. Thus, the next step is not simply to generate more high-dimensional data, but to convert these data into validated, scalable, and clinically actionable biomarkers that can guide treatment selection, timing, and adaptive therapeutic adjustment in real-world NSCLC populations.

## 8 Conclusion

Immune resistance in NSCLC arises from an interconnected network of antigen-presentation defects, IFN signaling dysregulation, suppressive TME remodeling, epigenetic reprogramming, and T cell dysfunction. Future therapeutic strategies should therefore move beyond single-target interventions toward integrated approaches that enhance antigen visibility, restore effector T cell function, reprogram the tumor microenvironment, and dynamically monitor tumor evolution during treatment. Although emerging technologies such as single-cell sequencing, spatial transcriptomics, and artificial intelligence may improve resistance monitoring, their clinical implementation will require standardized assays, prospective validation, interpretable models, and evidence that they can meaningfully improve treatment decisions.
